# Peptidases: Role and Function in Health and Disease

**DOI:** 10.3390/ijms24097823

**Published:** 2023-04-25

**Authors:** Janko Kos

**Affiliations:** Department of Biotechnology, Faculty of Pharmacy, Jožef Stefan Institute, University of Ljubljana, 1000 Ljubljana, Slovenia; janko.kos@ffa.uni-lj.si

Peptidases represent a large family of hydrolases present in all living organisms, which catalyze the degradation of peptide bonds in different biological processes [1]. In total, 2% of all protein-coding genes encode peptidases and their homologues in all kinds of organisms, and there are almost 600 active and putative peptidases in the human genome. Peptidases are involved in the degradation of off-function proteins in lysosomes, cytosol, plasma membranes, or in extracellular space; however, they may also have regulatory roles controlling biological processes crucial for cell homeostasis. In addition to being involved in normal protein turnover, their irregular function has been associated with a number of pathological processes, including cancer, neurodegenerative, immune and cardiovascular disorders, rheumatoid arthritis, osteoarthritis, atherosclerosis, periodontitis, pancreatitis, osteoporosis, diseases of the insufficient lysosomal degradation of proteins, and more. In view of the recent COVID-19 pandemic, the function of peptidases in viral uptake and replication has been exposed, and several approaches to targeting viral or host peptidases are suggested as tools for the prevention and treatment of disease. In this Special Issue, Geiger et al. [2] present a novel pyridyl indole ester and peptidomimetics as potent inhibitors of the severe acute respiratory syndrome coronavirus type 2 (SARS-CoV-2)’s main protease. In the paper, they analyzed the impact of these compounds on viral replication and demonstrated that they act in a cell-line-specific way. They further investigated three compounds in human precision-cut lung slices and observed donor-dependent antiviral activity. The results show that not only host cell proteolytic profile but also the sensitivity of viral peptidases for inhibition determine the viral uptake and replication in certain cell types.

In addition to viral infection and promotion, the peptidases are involved in several other parasites, such as the protozoan *Trypanosoma brucei rhodesiense*, which causes Human African Trypanosomiasis, also known as sleeping sickness, leading to meningoencephalitis. The cathepsin L-like cysteine peptidase in the parasite is involved in the penetration of the blood–brain barrier, and its activity is modulated by the chagasin-family endogenous inhibitor of cysteine peptidases (ICP). By using *CP*-null (Δ*icp*) mutants and wild-type strains, Costa et al. [3] demonstrated that ICP plays a pivotal role in *T. b. rhodesiense*, allowing the parasite to suppress host vasculature activation, myeloid cell recruitment, and the production of inflammatory cytokines with consequences to parasite fitness and survival.

Interestingly, cathepsin L-like peptidase was also found in pest insects from the family of *Tenebrionidae*, representing 72% of the total expression level of cysteine peptidase genes in the insect larvae gut. Cathepsin L (NCBI ID NP_001164001) (TcCathL1) appears to be the main cysteine digestive peptidase in *T. castaneum* and plays an important role in the initial steps of food protein digestion [4], including gluten proteins (gliadins) of wheat rich in proline and glutamine. Dvoryakova et al. [5] describe the expression of cathepsin L as a proenzyme (rpTcCathL1) and its processing to the mature enzyme and provide a detailed characterization of the mature enzyme’s properties and its ability to efficiently hydrolyze different immunogenic gliadin peptides. They propose cathepsin L as a drug candidate for the enzyme therapy of various types of gluten intolerance.

Additional pest peptidases, i.e., proline-specific peptidases (PSPs) in the midgut of the larvae of agricultural pests *Tenebrio molitor* and *Tribolium castaneum*, have also been proposed as candidates for the enzymatic therapy of celiac disease and gluten intolerance by Tereshchenkova et al. [6].

Bacterial peptidases represent a large group of enzymes with a high potential in biotechnology, food industry, and crop protection. In most cases, their 3D structure as well as their detailed functions still remain unknown. In this Special Issue, Petrenko et al. present the crystal structure of bacterial oligopeptidase B from *Serratia proteamaculans* (SpOpB) in a complex with a chloromethyl ketone inhibitor and discuss the similarities and differences between protozoan and bacterial enzymes [7]. Furthermore, Li et al. [8]. present the role of bacterial ε-PL-degrading enzyme (pldII) on the antibacterial effect of ε-Poly-L-lysine (ε-PL). ε-PL is a widely used antibacterial peptide polymerized of 25–35 L-lysine residues. The antibacterial effect of ε-PL is closely related to the polymerization degree. The authors utilized the integrative plasmid pSET152-based CRISPRi system to transcriptionally repress the pldII ε-PL and showed that its repression improves the antibacterial effect of the ε-PL product.

Three papers, selected for publication in this Special Issue, highlight new issues in blood coagulation and fibrinolysis cascades, classical topics of peptidase investigations. In a review paper, Plawinski et al. [9] present the mechanisms of plasminogen reception and activation at the surface of cell-derived microvesicles, and new actors in fibrinolysis and proteolysis. Microvesicles therefore provide a catalytic surface for plasmin generation potentially relevant in pathological settings, such as inflammation, atherosclerosis, angiogenesis, and tumor growth. In atherosclerotic plaques, the plasmin generation on macrovesicles could regulate the cell apoptosis/angiogenesis balance, influencing the plaque vulnerability. The question arises whether profibrinolytic microvesicles are in an equilibrium with pro-coagulant microvesicles, ensuring a balanced hemostasis, leading to the maintenance of vascular patency.

The homeostasis of the coagulation–fibrinolysis system is based on a delicate balance between proteases and their activators and inhibitors. As shown by Pablo-Moreno et al. [10], one molecule, such as coagulation factor V, can perform both a procoagulant and an anticoagulant function. The authors explained the dual role of factor V and stressed that the discovery of cost therapies of factor V deficiency has stretched out over too many years.

Factor-VII-activating protease (FSAP) is another serine peptidase involved in the regulation of hemostasis and inflammation. Extracellular histones are involved in the conversion of latent pro-FSAP into active FSAP, which has been shown, among other functions, to also regulate endothelial permeability. Cui et al. [11] investigated whether FSAP neutralizes the permeability-related effects of histones released upon tissue injury or inflammation and explored the effect of the serine protease domain (SPD) of FSAP on histone-induced endothelial permeability in vitro. The effect of the wild-type (WT)–SPD–FSAP was compared to the inactive MI–SPD–FSAP, as well as the role of TLR-2 and -4. Histones upregulated the expression of TLR-2, but not TLR-4, in HUVEC cells, and WT–SPD–FSAP abolished the upregulation of TLR-2 expression. The inhibition of histone-mediated permeability may be an important function of FSAP with relevance to sepsis, trauma, and stroke.

Two further review papers are included in this Special Issue. The first [12] is focused on the role of legumain in the regulation of biological processes and in the pathogenesis of various malignant and nonmalignant diseases, including cancer, bone remodeling, cardiovascular and cerebrovascular diseases, fibrosis, aging and senescence, and neurodegenerative diseases. The second [13] describes SUMO modification as one of post-translational regulation processes in eukaryotes. In this process, SUMO protease is responsible for the maturation of the SUMO precursor and the deconjugation of the SUMO protein from modified proteins by cleaving behind the C-terminal Gly–Gly motif. The authors systematically analyzed the specificity of the *S. cerevisiae* SUMO protease (Ulp1) on the cleavage of the C-terminal motif.

To summarize, this Special Issue presents only a small view of the research activities on the role and function of the proteolytic system in physiological processes and on their harmful functions in diseases; however, it uncovers the complexity of protein degradation and highlights the need of further extensive studies to fully understand proteolytic processes. The new knowledge can strengthen the potential of these enzymes as targets for the development of new diagnostic and therapeutic tools for the better treatment of a variety of related diseases.
